# Characterization of Polyphenol Composition and Starch and Protein Structure in Brown Rice Flour, Black Rice Flour and Their Mixtures

**DOI:** 10.3390/foods13111592

**Published:** 2024-05-21

**Authors:** Alexandra Uivarasan, Jasmina Lukinac, Marko Jukić, Gordana Šelo, Anca Peter, Camelia Nicula, Anca Mihaly Cozmuta, Leonard Mihaly Cozmuta

**Affiliations:** 1 Department of Chemistry-Biology, Technical University of Cluj Napoca, 430122 Baia Mare, Romania; uivarasan.al.alexand@student.utcluj.ro (A.U.); anca.peter@cb.utcluj.ro (A.P.); camelia.nicula@cb.utcluj.ro (C.N.); mihaela.mihaly@cb.utcluj.ro (A.M.C.); 2 Faculty of Food Technology, Josip Juraj Strossmayer University of Osijek, 31000 Osijek, Croatia; jlukinac@ptfos.hr (J.L.); marko.jukic@ptfos.hr (M.J.); gordana.selo@ptfos.hr (G.Š.)

**Keywords:** rice, FTIR spectra deconvolution, starch crystallinity, amide I band in proteins, color analysis, reflectance spectra deconvolution

## Abstract

The study investigates the structural and chemical properties of brown rice flour (WRF), black rice flour (BRF) and their mixtures in ratios of 25%, 50% and 75% to provide reference information for the gluten-free bakery industry. BRF contains higher concentrations of proteins, lipids, total minerals, crude fiber, total polyphenols, proanthocyanidins and flavonoids than WRF. A higher amylose content in BRF than in WRF resulted in flour mixtures with slower starch digestion and a lower glycemic response depending on the BRF ratio added. Differences in the chemical composition of WRF and BRF led to improved composition of the flour mixtures depending on the BRF ratio. The presence of anthocyanidins and phenolic acids in higher concentrations in the BRF resulted in a red–blue color shift within the flour mixtures. The deconvoluted FTIR spectra showed a higher proportion of α-helixes in the amide I band of BRF proteins, indicating their tighter folding. An analysis of the FTIR spectra revealed a more compact starch structure in BRF than in WRF. By processing reflection spectra, nine optically active compound groups were distinguished in rice flour, the proportion in BRF being 83.02% higher than in WRF. Due to co-pigmentation, the bathochromic shift to higher wavelengths was expressed by the proanthocyanins and phenolic acids associated with the wavelengths 380 nm to 590 nm and at 695 nm. Anthocyanins, protein–tannin complexes, methylated anthocyanins and acylated anthocyanins, associated with wavelengths 619, 644 and 668 nm, exhibited a hypsochromic effect by shifting the wavelengths to lower values. This research represents a first step in the development of rice-based products with increased nutritional value and a lower glycemic index.

## 1. Introduction

Rice (*Oryza sativa* L.) is considered the most important crop in the world, given its global production and consumers [1]. Due to its gluten-free nature, it is often used in gluten-free diets. However, derived white rice-based food products have a drawback in terms of a high glycemic index, which could increase insulin resistance in people who consume large amounts of these. Given the growing need to develop functional foods, research has been conducted to find alternative rice sources richer in slowly digestible starch, which could lower the glycemic index during digestion [2]. The whole grains attracted attention due to their invaluable nutritional value, which can improve the functionality of traditional white rice-based products. A higher amount of minerals and fibers and the presence of phenolics, vitamins, γ-oryzanols, tocopherols, tocotrienols and phytosterols located in the bran layer of the rice grain are considered health-promoting in terms of antioxidant and anti-inflammatory properties [3,4]. Even phytic acid, commonly considered an antinutrient because it complexes with minerals and proteins and reduces their bioavailability, is thought to have some medicinal properties in preventing certain types of cancer [1]. Polyphenols are considered to be the major class of bioactive compounds with antioxidant properties in rice, with phenolic acids predominating in white rice and procyanidins in red rice [4]. The characteristic polyphenols in black rice are mainly flavanoids and anthocyanins. Flavanoids, generally occurring as O- or C-glycosides and divided into flavones, flavanols, flavanones, flavonols and iso- flavone, inhibit lipid peroxidation and impart antioxidant activities [3]. A number of seven flavonoids, represented by tricin (77%), luteolin (14%), apigenin (6%), quercetin (3%), isorhamnetin (1%), kaempferol (<1%) and myricetin (<1%), were predominantly found in the bran of black rice [5]. Anthocyanins, the major bioactive and functional compounds in black rice bran, represent a group of reddish-to-purple water-soluble flavonoids. They are mostly found in the form of polyhydroxylated or methoxylatedheterosides, derived from the flavylium ion or 2-phenyl benzopyrilium [6]. Among them, cyanidin-3-glucoside, peonidin-3-glucoside, cyanidin-3,5-diglucoside and cyanidin-3-rutoside account for a large proportion [7]. In addition to flavanoids and anthocyanins, black rice contains phenolic acids, some of which are in a conjugated soluble form (cinnamic acid, protocatechuic acids, gallic acids), while others are covalently bound (ferulic acid, coumaric acid, caffeic acid) with cellulose, hemicellulose or lignin [6]. Black rice is richer in minerals (Ca, P, Fe and Zn) and fiber than brown and white rice, making it a nutrient-dense food [8], which can lead to a feeling of satiety faster, while reducing food intake. Tocopherols, with higher concentrations in α tocopherol and oryzanols than in red and white rice, exhibit high antioxidant properties, contribute to a reduction in blood plasma and serum cholesterol, hyperlipidemia and platelet aggregation and improve uterine health by treating menopausal disorders [5]. Due to its various bioactive compounds and their high concentrations, black rice has great potential for use in the production of functional foods and drinks. The use of black rice in the food industry requires information about its structural and functional properties. The foaming properties, oil holding and swelling capacity, emulsifying properties, glycemic index, digestibility and the behavior of rice flour during the preparation, consumption or storage of rice-based foods are significantly influenced by the starch and protein structure. The information in the literature on the protein and starch structure in rice is rather poor [9]. The quantification of individual polyphenols in rice flour is a time-consuming analysis and requires high costs for instrumentation and reagents. Furthermore, it does not provide insights into the co-pigmentation between the anthocyanins themselves or with other molecules that lead to the hyperchromic properties of rice flour and color changes due to bathochromic or hypsochromic effects [10]. The reflectance spectra has been successfully used to predict the protein, amylose, total polyphenol and fiber content in rice or its antioxidant activity [11]. No data have been found in the literature on the application of reflectance spectra to identify the optically active compound groups and their co-pigmentation, which could help explain some of the sensory properties of rice flour, such as astringency and bitterness, or some redox properties, such as the antioxidant activity.

From this perspective, the aim of the paper is to comparatively assess the composition, structure and chemical parameters of brown rice flour, black rice flour and their mixtures as a first step in the development of rice-based products with increased nutritional value and a lower glycemic index. Thus, reference information is provided for the gluten-free bakery industry to pave the way for the industrial production of black rice-based food.

## 2. Materials and Methods

### 2.1. Raw Materials and Reagents 

Black rice grains (BRF) and brown rice grains (WRF) were purchased from a local supermarket. The grains were examined for foreign material and broken particles, ground into powder using a laboratory mill (IKA Labortechnik MF 10, IKA-Werke GmbH & Co. KG, Staufen im Breisgau, Germany) and sieved through a 1 mm sieve. Flour mixtures were prepared with 25% (25-BRF), 50% (50-BRF) and 75% (75-BRF) black rice flour proportions. Hydrochloric acid, sodium hydroxide, ethanol, acetic acid, iodine solution, methanol, hexane, sodium carbonate, Folin-Ciocalteu reagent, diethyl ether, ethyl acetate, rice amylose, butanol, sodium nitrite, aluminum chloride, iron sulphate, formic acid and acetonitrile were purchased from Merck (Rahway, NJ, USA). Quercetin, gallic acid, cyanidins and standards for the U-HPLC analysis of phenolic acids and anthocyanins were analytical grade and were obtained from Sigma Aldrich (Saint Louis, MO, USA). The glucose oxidase/peroxidase D-glucose assay kit was purchased from Biosystems (Barcelona, Spain).

### 2.2. Characterization of Rice Flours 

#### 2.2.1. Proximate Analysis

The standard chemical methods were applied to determine the dry matter content, the content of total proteins, total lipids, ash and total fibers [12,13,14,15]. The total starch content in flour was measured based on its hydrolysis to maltodextrins by α-amylase and subsequent hydrolysis to D-glucose in the presence of amyloglucosidase [16]. D-glucose is oxidized to D-gluconate with the release of H_2_O_2_, which is measured quantitatively at 510 nm in a colorimetric reaction with peroxidase, producing quinonimine. Based on the results of the proximal analysis, the carbohydrate content and energy provided by each sample were determined.

#### 2.2.2. Total Amylose Analysis

The iodine-binding method was used to quantify the amylose content of rice flour [17]. A 100 mg amount of sample was mixed thoroughly with 1 mL of ethanol (95%) and 9 mL of 1 N NaOH. The solution was heated in a boiling water bath for 10 min to gelatinize the starch and then allowed to cool to room temperature. A volume of 5 mL of gelatinized starch solution was transferred to a 100 mL volumetric flask, mixed with 1 mL of 1 N acetic acid and 2 mL of iodine solution and diluted to 100 mL using distilled water. After stirring for 20 min, the absorbance was measured at 620 nm (Perkin Elmer Lambda 35 UV-VIS spectrophotometer). A standard curve based on rice amylose was used to quantify the amylose content of the sample. 

#### 2.2.3. FTIR Analysis

A Perkin Elmer spectrometer BX2 (Waltham, MA, USA) with a Pike Miracle ATR diamond crystal was used to collect spectra in the wavenumber range of 600 to 4000 cm^−1^. The flour samples were placed directly on the crystal and scanned under the same pressure in 50 repetitions with a resolution of 8 cm^−1^. A total of 10 spectra were collected from each flour type. The spectra were baseline corrected and normalized in the range 0 to 1 to remove the contribution of background noise, interference caused by radiation scattering or certain intrinsic fluorescence signals of the samples. Therefore, the adsorption variation was attributed only to the chemical properties of the samples. 

The spectra in the range of 1600–1700 cm^−1^ correspond to the secondary structure of the amide I band of the protein and are related to the vibration of the backbone structure, mainly the C=O stretching vibration of the amide groups coupled with some in-plane N-H bending. It was deconvoluted through an iterative fitting protocol assuming the Gaussian band form. First, a Savitzky–Golay filter (polynomial degree = 3, points = 9) was applied to each spectrum, and the positions of the band centers were determined using second derivative analysis. The relative areas of these bands were used to estimate the percentage of secondary structural features: β-turns (1660–1700 cm^−1^), α-helixes (1650–1658 cm^−1^), random coils (1640–1650 cm^−1^) and β-sheets (1600–1640 cm^−1^) [18].

The crystalline/amorphous structure of starch in the rice flours was examined in the range of 900–1200 cm^−1^ [18]. A similar protocol was applied as in the case of the protein assay study: baseline correction, Savitzky–Golay filtration leading to a second derivative for which the minimum values correspond to the positions of the band centers. Based on previously determined wavenumbers, the characteristic parameters of each distribution were determined to minimize the differences between the base spectrum and the sum of the individual distributions. The short-range crystal structure in rice starch was calculated as the ratio of the peak areas at 1047 cm^−1^ and 1022 cm^−1^ (R_1047/1022_). The higher R_1047/1022_, the higher the degree of order in the starch structure. The ratio of the peak areas at 995 cm^−1^ and 1022 cm^−1^ (R_995/1022_) was selected to describe the structure of hydrogen bonding between the starch molecules (the double helix degree of the starch structure) [19].

#### 2.2.4. Extraction and Quantification of Total Polyphenols

The rice flour was *defatted* according to the protocol described by Shamanin et al. [20] adapted to our work. The flour sample was mixed with hexane (1:5 w:v), stirred at 200 rpm for 10 min (Arex-6 hot plate stirrer, Velp Scientific Inc., New York, NY, USA) and centrifuged at 2500× *g* for 5 min (laboratory digital centrifuge, Petria Life Science LLP, Bengaluru, India). The supernatant was removed, and the solid phase was mixed again with hexane in the initial ratio and centrifuged. The extraction was repeated three times, and the defatted samples were dried on a filter paper under the hood for 12 h.

The *free phenolic compounds* were extracted according to the method proposed by Jang and Xu [21] adapted to our work. An accurately weighed 5 g of defatted sample was extracted with 30 mL of 80% aqueous methanol under ultrasound treatment for 25 min at 25 °C (Bandelin Sonorex Super RK, BANDELIN electronic GmbH & Co. KG, Berlin, Germany, 35 KHz, 80 W). The mixture was centrifuged at 2500× *g* for 5 min, and the solid was subjected to similar extractions two more times. The resulting supernatants containing free phenolic compounds were pooled and subjected to analysis. 

Total phenolic compounds were extracted from 5 g of defatted sample with 50 mL of 95% ethanol at room temperature at 200 rpm for 6 days [22]. After filtration, the solution was analyzed.

To analyze polyphenol content, a volume of 2 mL of extract was put in contact with 0.5 mL of 20% Folin–Ciocalteu reagent and allowed to react for 5 min, and another 0.5 mL of saturated Na_2_CO_3_ solution and 5 mL of deionized water were added. The solution was kept in the dark for 2 h, and the absorbance was read at 765 nm (Perkin Elmer Lambda 35 UV-VIS spectrophotometer). The gallic acid-based calibration curve was used to quantify the phenolic compounds. The results were expressed as mg gallic acid equivalent (GAE)/100 g sample.

The *bound phenolic* content was calculated as the difference between the total polyphenols and the content of free polyphenols. 

#### 2.2.5. Extraction and Quantification of Total Flavonoids and Total Proanthocyanidins

All samples were subjected to extraction by mixing 1.5 g of rice flour with 20 mL of 70% aqueous ethanol at 80 °C, in a water bath at 200 rpm. After 1 h, the sample was centrifuged at 10,000× *g* for 10 min, and the supernatant was collected. The extraction of solid residue was repeated three times and the supernatants were combined and further used for the analysis of total flavonoid and total proanthocyanidins.

*Total flavonoid content* (TFC) was measured by following the method proposed by Marinova et al. [23]. An exact volume of 0.5 mL was mixed with 2 mL of distilled water, 0.15 mL of 0.5% sodium nitrite (w:v) and 0.15 mL of 10% aluminium chloride (w:v). After 6 min of the reaction, a volume of 1 mL of 1 M NaOH was added and the mixture was diluted with 1.2 mL distilled water. The absorbance of the final solution was read at 510 nm after 15 min. The results were expressed as (+) mg quercetin equivalent (QE) per gram of dried rice flour (mg_QE_/g).

The *total proanthocyanidin* content (TPA) was determined by using the method proposed by Škerget et al. [24]. A volume of 50 mL of the extract was mixed with 20 mL of FeSO_4_ solution (77 mg of FeSO_4*_•7H_2_O in 500 mL of HCl:n-butanol 2:3 v:v). After 15 min of stirring at 95 °C on the water bath, the mixture was cooled under water, and the absorbance was read at 540 nm against a blank containing distilled water instead of the sample. The results were expressed as mg cyanidin/g of dried sample (mg_C_/g).

#### 2.2.6. Quantification of Individual Polyphenols

The identification and quantification of selected polyphenols were carried out using ultra-high-performance liquid chromatographic (U-HPLC) analysis (U-HPLC Nexera XR, Shimadzu). The extracts for the U-HPLC analysis of phenolic compounds were prepared according to the protocol described in Section 2.2.5. The separation was achieved with a Kinetex^®^ C18 column (100 × 4.6 mm, 2.6 μm, Phenomenex, Torrance, CA, USA) equipped with a photodiode (PDA) detector. Prior to U-HPLC analysis, the samples were filtered through 0.45 μm membranes. The software LabSolutions 5.87 was used to process experimental data.

Two mobile phases were used for the analysis of *proanthocyanidins* [25]. The gradient mobile phase A consisted of water:formic acid:acetonitrile (87:10:3 v:v:v). The gradient phase B was made of water:formic acid:acetonitrile (40:10:50 v:v:v), and the chromatographic gradient was as follows: 10 min solvent B from 10% to 25%, 5 min solvent B from 25% to 31%, 5 min solvent B from 31% to 40%, 10 solvent B min from 40% to 50%, 10 min solvent B from 50% to 100% and 10 min solvent B from 100% to 10%. A volume of 20 μL of sample was injected into the column at a flow rate of 0.8 mL/min. The quantification of proanthocyanidins was performed at 513–531 nm, using individual calibration curves. 

The analysis of *phenolic acids* used 20 μL of sample injected, coupled with two phases of the linear gradient. Phase A consisted of 1.0% aqueous acetic acid (v:v), while phase B was made of methanol:acetonitrile (50:50 v:v). At a flow rate of 1 mL/min and 30 °C, a linear gradient was achieved as follows: 25 min for B solvent from 5% to 30%, 10 min for B solvent from 30% to 40%, 5 min for B solvent from 40% to 48%, 10 min for B solvent from 48% to 70%, 5 min for B solvent from 70% to 100%, 5 min isocratic solvent B at 100%, followed by the return to initial conditions in 10 min and another 10 min for column equilibration. By comparing the UV-VIS spectra and retention times of standards analyzed under the same conditions, individual phenolic compounds were detected and quantified using calibration curves generated with external standards [26].

#### 2.2.7. Color Analysis

The color of rice flour was assessed at 25 °C using a YL 4560-3nh non-contact benchtop spectrophotometer (Shenzhen Threenh Technology Co., Ltd., Shenzhen, China) and expressed as CIEL*a*b* color coordinates. The instrument was calibrated against standard white and black tiles before each measurement. 

#### 2.2.8. Reflectance Spectra

The reflectance spectra of the flours were recorded with the Perkin Lambda 35 UV-VIS Spectrophotometer equipped with a Labsphere RSA-PE-20, integrating a sphere with a resolution of 1 nm over the spectral range of 360–780 nm. Five repetitions were conducted for each flour type. For each wavelength, the means and standard deviations corresponding to these five reflectance values were calculated. The average of the mean values (AMV) and the average of the standard deviations (ASD) were calculated for the entire spectral range considered. The relative standard deviation RSD (%) was calculated as the percentage ratio of ASD to AMV. For all rice flours investigated, the RSD (%) values were situated in the range of 0.2263–1.3513%, which highlights the quality of the measurements and the high homogeneity of the samples. 

### 2.3. Statistical Analysis

Each experiment was conducted in at least three replicates, and the results were expressed as the mean ± standard deviation. For statistical analysis, the one-way analysis of variance (ANOVA) test was used at a significant level of *p* < 0.05 to show whether there is a statistically significant difference between more than two different sets of similar data (composition, color and structure values for different samples) that caused the variability in the data. The Tukey test was used to compare the average values of each data set with the average values of the other sets and to identify any statistically significant difference (*p* < 0.05) greater than the global standard error characteristic of all groups, determined based on ANOVA analysis (considering the number of individual data points in each group and the remaining mean squares). The value of the Pearson correlation coefficient was used to assess the degree of linearity between two experimental variables (one is considered independent and the other one is considered linear dependence). Based on the simple linear regression model, the modeled values of the dependent variables were obtained using the least squares method, and the correlation coefficient between the experimental and modeled values of the dependent variables was calculated. The magnitude of the linear difference between the two variables increases with the correlation coefficient value. This algorithm revealed a linear dependence (similar to the Lambert–Beer law) between the absorbance values (obtained via Kubelka–Munk transformation) and the mixing ratio of the two types of rice flour. This dependence does not follow a linear relationship between the reflectance values and the flour mixing ratios (evidenced by the lower value of the correlation coefficient). Similar to the logarithmic relationship between the transmittance and concentration, reflectance and flour mixing ratios also present a logarithmic dependence. Spectral deconvolution was used to determine the secondary structures of proteins, the crystalline and amorphous structure of starch in flours and the classes of optically active color compounds. Section 2.2.3 and Section 3.6 provide a detailed description of the methodology used.

## 3. Results

### 3.1. Proximate Composition of Rice Flours

The proximate compositions of the flours and corresponding flour mixtures are displayed in Table 1. Concentrations of 1.26-, 1.90-, 1.89- and 2.01-times higher of total proteins, lipids, total mineral elements and crude fiber were obtained for the black rice flour as compared to brown rice flour. The present results are close to those reported by Banu and Aprodu [27]. Although the examined rice flours were characterized by significantly different amounts of carbohydrates, proteins, fiber and lipids, no statistically significant differences could be found between the energy provided by WRF, BRF or flour mixtures. From a commercial perspective, BRF is classified as having a medium-amylose content with an amylose content of more than 20%, while WRF (with 19.16%) is classified as have a low-amylose content. 

Amylose content is important because it affects the texture of cooked rice, particularly firmness and stickiness, two properties that influence consumer preference. The amylose concentration was 16.39% higher in BRF than in WRF. The result is far higher than the value of 6.47% reported by Farooq et al. [28], whereby parameters, such as environmental variables (temperature, location, light, biotic stresses), the cultivar, cultural practices (irrigation, fertilization, harvesting) or processing conditions could have been considered responsible [29]. The work of Brunet-Loredo et al. [30] reported a 29.9% increase in the polyphenol content in rice cultivars exposed to water-deficit conditions compared to rice varieties obtained in flooded grains. The cause is related to water-stress conditions, which promoted the excessive accumulation of oxygen reactive species in plant cells and led to damage to DNA, proteins and membranes. The increase in the polyphenol content may represent an acclimation response due to the antioxidant activity of polyphenols. Soil nutrients also affected the mineral content of rice. Under stress conditions, higher concentrations of Mg, Ca, Fe, Mn and Cu were found in black rice than in white rice [31]; specific enzymes are involved in scavenging free radicals, which use metals as electron acceptors in the antioxidant processes being activated [32]. When rice was exposed to an increased CO_2_ level, the flavonoid content decreased by 25% in the case of apigenin in white rice and by 12% in the case of bran [33]. The same study found a 35%, 32%, 25% and 2% decrease in *γ*-oryzanol in white rice, brown rice, husk and bran, respectively. Tocopherols and tocotrienols in white and brown rice were also reduced by 69% for *α*-tocotrienol, 46% for *γ*-tocotrienol and 38% for *α*-tocopherol. 

### 3.2. FTIR Spectroscopic Analysis of Rice Flours

**Figure 1:**
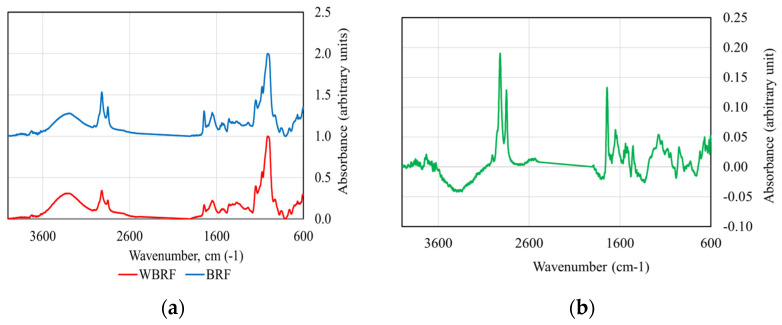
FTIR spectra of brown rice flour and black rice flour (**a**) and the differences between the average spectra (**b**).

The average spectra for WRF and BRF are shown in Figure 1a, and the differences between the average spectra are shown in Figure 1b.

The positions of the characteristic peaks corresponding to the maximum absorbance of proteins, polysaccharides and lipids are listed in Table S1 in the Supplementary Material. The data analysis shows that black rice flour has higher adsorption maximum values than brown rice flour, for the same starch content. Table S2 in the Supplementary Material summarizes the absorption ratios extracted from FTIR spectra. Average ratios of 1.63 for lipids, 1.16 for proteins and 1.05 for polysaccharides indicate higher amounts of lipids and proteins in BRF compared to WRF and almost equal concentrations of polysaccharides in both types of flours. These findings are supported by the results of the proximate analysis (Table 1).

**Figure 2:**
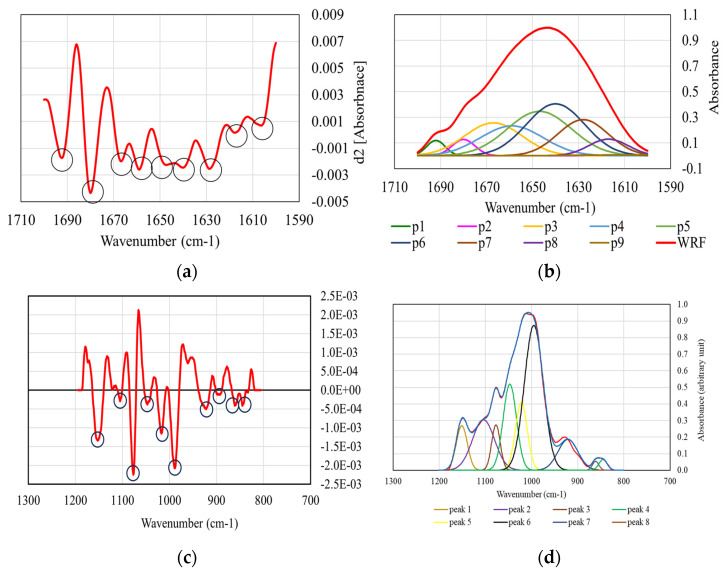
The second derivative of the FTIR spectrum for the amide I band in the protein association of WRF (**a**) and the deconvoluted curves (**b**); the second derivative of the FTIR spectrum for the starch band associated with WRF (**c**) and the deconvoluted curves (**d**).

Figure 2a presents the shape of the second derivative of the FTIR spectrum for the amide I band in the protein associated with WRF, while Figure 2b shows the deconvoluted curves that resulted from the above shape. Second-order derivatives were presented to show that wavenumbers corresponding to minimum values were used as centers of the Gaussian distributions in the deconvolution process. The relative areas of the bands from Figure 2b were used to estimate the percentage of the secondary structural features, and the results are presented in Table 2. They show a significantly higher proportion of α-helixes in BRF (20.47%) compared to WRF (14.22%), indicating a tighter folding of the proteins and implying a more ordered structure. The same trend was observed for the β-sheet, the amount being higher in BRF (32.40%) than in WRF (30.526%). The β-sheet structure is mainly stabilized by hydrogen bonds between the amino groups (-NH) and carbonyl groups (-CO) and plays an important role in the development of aggregate structures due to its relatively large surface area. No significant differences were found between the disordered random coil structure values. The disordered protein molecules, due to the unfolding of the α-helices, give the molecular structure more flexibility and expose the hydrophobic groups on their surface, which can lead to poor gelatinization and functional properties of the starch. Values of 36.9% for random coils and α-helixes, 20.9% for β-turns and 42.2% for β-sheets were reported by Solaesa et al. [9] for rice flour. No data on the secondary structure of proteins in black rice flour were found in the literature. 

Figure 2c displays the shape of the second derivative of the FTIR spectrum for the starch band associated with WRF, while Figure 2d presents the deconvoluted curves. It can be noticed that the peaks assigned to wavenumbers 995 cm^−1^, 1022 cm^−1^ and 1047 cm^−1^ (marked as p6, p5 and p4 in Figure 2d) correspond to the central region of the spectrum marked by the highest absorption value. The R_1047/1022_ and R_995/1022_ indices used in the assessment of the short-range/double helix order of starch are shown in Table 3a,b. Their values were significantly higher in BRF (R_1047/1022_ = 1.32, R_995/1022_ = 1.57) compared to those obtained for WRF (R_1047/1022_ = 1.12, R_995/1022_ = 1.32), which indicates a more ordered starch structure due to the larger amount of double helix chains responsible for the arrangement of starch molecules in a compact way. The work of Solaesa et al. [9] reported values of 0.717 and 0.865 for R_1047/1022_ and R_995/1022_ indices in brown rice flour. The starch structure significantly influences its gelatinization, which changes the texture and palatability of the products. Gelatinization occurs in the presence of moisture and heat and begins in the amorphous region of the starch, where the hydrogen bonds are weaker. The larger the amorphous region, the lower the gelatinization temperature. Larger pores formed in the amorphous internal structure facilitate the water penetration and increase the swelling capacity. In a compact region, the formation of large pores is not allowed and water access is restricted. This results in a diminished swelling capacity and starch solubility [34]. Apart from the gelatinization process, the starch retrogradation is also influenced by its molecular structure. The gelation of solubilized amylose during gelatinization and the recrystallization of amylopectin within the gelatinized granules are the main processes that occur during cooling and storage of starch. Due to a lower water permeability in the compact region of the starch, the amount of amylose leaching is reduced and the retrogradation of the starch can be slowed down. Therefore, slower aging of products based on black rice is expected, compared to brown rice flour. Increasing the order structure of starch leads to reduced digestibility and glycemic indexes, as demonstrated by the digestion study in the Supplementary Material (Figures S1 and S2, Table S3). 

### 3.3. The Contents of Total Polyphenols, Total Flavonoids and Total Proanthocyanidins

Table 1 lists all of the polyphenolic compounds present in the flours and their combinations. The findings indicated that the amounts of total polyphenols (58.05%), free polyphenols (34.06%) and bound polyphenols (73.96%) in black rice flour are higher than in brown rice flour. Previous studies reported that the pigmented rice varieties generally have significantly higher total polyphenol contents than the non-pigmented rice varieties. Huang and Ng [35] reported that the total polyphenols content ranged from 0.24 to 0.45 g GAE/Kg in non-pigmented brown rice. The work of Kong and Lee [36] found 16.4 mg GAE/g of sample for free polyphenols, 1.76 mg GAE/g of sample for bound polyphenols and 18.2 mg GAE/g of sample for total polyphenols in *Oryza sativa* cv. *Heugjinjubyeo. Oryza sativa* cv. *Heugjinjubyeo* showed a concentration of 11.8 mg GAE/g of sample for free polyphenols, 1.53 mg GAE/g of sample for bound polyphenols and 13.32 mg GAE/g of sample in the case of total polyphenols. In our study, the bound phenolic compounds, which have higher antioxidant and antimicrobial activity in the colon than the free polyphenols [37], accounted for 36.97% in brown rice and 59.56% in black rice. The values for the total flavonoid content and total proanthocyanidins in the examined flour samples are provided in Table 1. Among flavonoids and proanthocyanidins, the first class exhibits the highest content in BRF and flour mixtures. The total content of flavonoids and proanthocyanidins in black rice flour was significantly higher (*p* < 0.05) than in brown rice flour with 88.18% and 99.33%, respectively. The addition of BRF resulted in the enrichment of 25-BRF, 50-BRF and 75-BRF by 2.19-, 3.56-, and 5.68-fold in flavonoids and 38.00-, 74.00-, and 116.00-fold in proanthocyanins. 

### 3.4. Quantification of Individual Polyphenols

Out of six anthocyanins previously reported to normally occur in black rice (cyanidin-3-*O*-glucoside, peonidin-3-*O*-glucoside, malvidin-3-*O*-glucoside, petunidin, myrtillin and pelargonidin-3-*O*-glucoside) [38], the first four were identified in the black rice flour sample (Table 3a). 

Cyanidin-3-*O*-glucoside and peonidin-3-*O*-glucoside predominated in black rice flour, with concentrations of 481.68 µg/g and 203.74 µg/g, while petunidin chloride was found at the lowest concentration of 1.07 µg/g. As Table 3a shows, the proanthocyanidin family is absent in brown rice flour. An analysis of variance revealed statistically significant increases (*p* < 0.05) in the proanthocyanidin content of 25-BRF, 50-BRF and 75-BRF upon the addition of BRF. The study of Goufo and Trindade [5] found concentrations of 54.59 mg/100 g and 93.42 mg/100 g for peonidin-3-*O*-glucoside and cyanidin-3-*O*-glucoside in brown rice grains. They also detected petunidin as a minor constituent in the form of petunidin-3-*O*-glucoside, petunidin-3-*O*-galactoside or petunidin-3-*O*-arabinoside.

Of 13 phenolic acids tested, seven phenolic acids were identified in black rice flour (Table 3b). Protocatechuic acid was the most abundant (205.80 µg/g), followed by vanillic acid (25.07 µg/g), ellagic acid (7.37 µg/g), syringic acid (6.03 µg/g), ferulic acid (3.59 µg/g), p-coumaric acid (0.89 µg/g) and sinapic acid (0.51 µg/g). Apart from protocatechuic acid and p-coumaric acid, the other phenolic acids presented lower concentrations in brown rice flour than in black rice flour, without maintaining the ranking observed in BRF. Ferulic acid is the main constituent of brown rice flour (4.84 µg/g), followed by syringic acid (1.13 µg/g), sinapic acid (0.34 µg/g), vanillic acid (0.27 µg/g) and ellagic acid (0.25 µg/g). Protocatechuic acid was not identified in the WRF.

In our study, p-coumaric acid has a higher concentration of 1.03 µg/g in WRF than in BRF (0.89 µg/g), which is in accordance with the study published by Goufo and Trindade [5], where the concentrations of 0.61 mg/100 g for p-coumaric acid were obtained for non-pigmented rice and 0.40 mg/100 g for pigmented rice. The 3.71-fold represents higher phenolic acid contents in BRF than in WRF and suggests higher antioxidant activity in BRF, due to the ability of the aromatic ring in the phenolic acids to act as a scavenger of oxygen-reactive species (R●) and interrupt the chain reactions. The result is consistent with the findings of Goufo and Trinidade [5], which indicated that among the four types of rice ranked by color, black rice varieties exhibited the highest antioxidant activities, followed by purple, red and brown rice varieties.

### 3.5. Color Analysis of Flours

Table 1 shows the influence of BRF addition on the color of the flour mixtures. Color parameters exhibited a decrease in lightness (L*) and yellowness (b*), accompanied by an increase in redness (a*) for the flour mixtures with higher ratios of BRF. Since their total concentration in BRF was higher than that of phenolic acids, cyanidin-3-*O*-glucoside, peonidin-3-*O*-glucoside, malvidin 3-*O*-glucoside and petunidin chloride, which impart a red–blue color, can be considered to be the primary cause of this color shift (Table 3a,b). The correlation coefficients between the color parameters and the BRF ratio of −0.9665 for L*, 0.9802 for a* and −0.8833 for b* were statistically different. 

No literature data were found on the influence of the addition of black rice flour on the color of a mixture of brown rice flour and black rice flour. 

### 3.6. Reflectance Spectra

**Figure 3:**
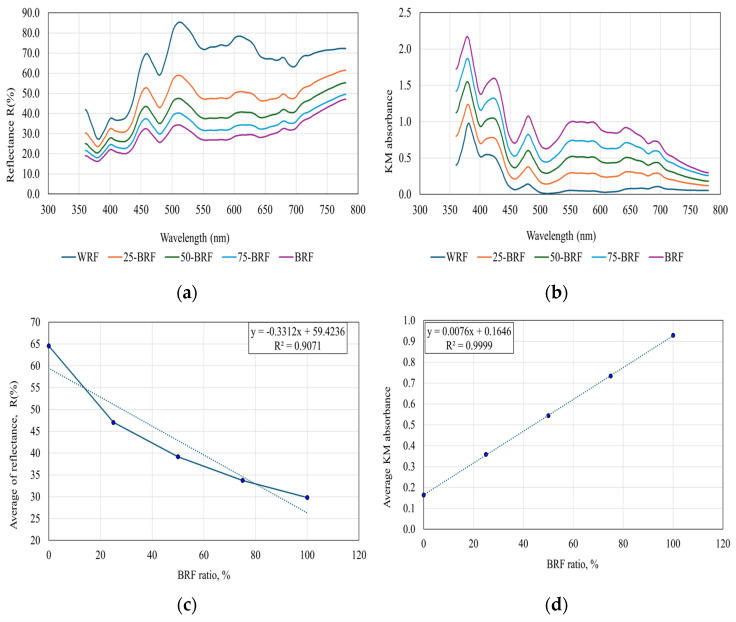
The processing of the reflectance spectra for the investigated flours: (**a**) the average reflectance spectra; (**b**) the variation in KM absorbance and spectral range wavelengths with the ratio of added BRF; (**c**) correlation between the raw reflectance spectra and the ratio of BRF added; (**d**) correlation between KM-transformed reflectance spectra and the ratio of added BRF.

Figure 3a shows the average reflectance spectra for the examined flours. Analyzing the data obtained, it can be observed that WRF reflects the most incident radiation, while BRF reflects the same radiation to the smallest extent. Considering the principle according to which a colored body will reflect the radiation corresponding to its own color and will reflect the other shades of color, the conclusion would be that WRF has a much higher content of colored compounds than BRF. This conclusion is in contradiction with the results of the chemical analysis (Table 3a,b); therefore, the color characterization of samples based on the reflection spectrum is not appropriate in this case. 

For an accurate assessment of the color of the analyzed flours, their raw reflectance spectra were transformed according to the Kubelka–Munk (KM) equation:

AC = k/s = (1 − R)^2^/2R (1)

where AC is the KM absorbance, k is the absorption coefficient, s represents the scattering coefficient and R is the reflectance.

The absorption coefficient (KM absorbance) is proportional to the concentration, while the scattering coefficient depends on the particle dimension and refractive index of the sample and is relatively independent of the wavelength. While maintaining a constant particle size, the absorbance determined using the Kubelka–Munk equation can be utilized to evaluate the absorbance of the sample in correlation with the concentration of optically active compounds in the analyzed sample. Figure 3b shows the variation in the KM absorbance and spectral range wavelengths with the ratio of BRF added. Each value is an average of five individual determinations based on the same sample. The dependences between the mean of the reflectance spectra and the mean of the absorbance values (KM) in all frequency ranges as a function of the BRF ratio are shown in Figure 3c,d. Their analysis indicates that a logarithmic dependence is expressed between the raw reflectance spectra and the BRF added ratio, while in the case of reflectance spectra transformed with the Kubelka–Munk equation, the dependence is linear. A correlation coefficient of −0.95241 indicates that as the BRF ratio increases, the mean reflectance decreases. In the case of KM transformation, a linear increase was observed with a correlation coefficient of 0.99997. As displayed in Figure 3b, the highest absorbance values were obtained for BRF, with the reduction of BRF in the flour mixtures, leading to a decrease in the absorbance values. According to the principle of absorption of complementary radiation, these variations indicate a decrease in the concentration of optically active compounds in samples according to the results of the chemical analysis (Table 3a,b).

Classes of differently colored compounds were differentiated based on the deconvolution of the KM absorption spectra. The aim was to calculate the areas of the Gaussian distributions that corresponded to the absorption maxima identified in the base spectra. The process, illustrated for the 50-BRF sample, consisted of two steps. 

**Figure 4:**
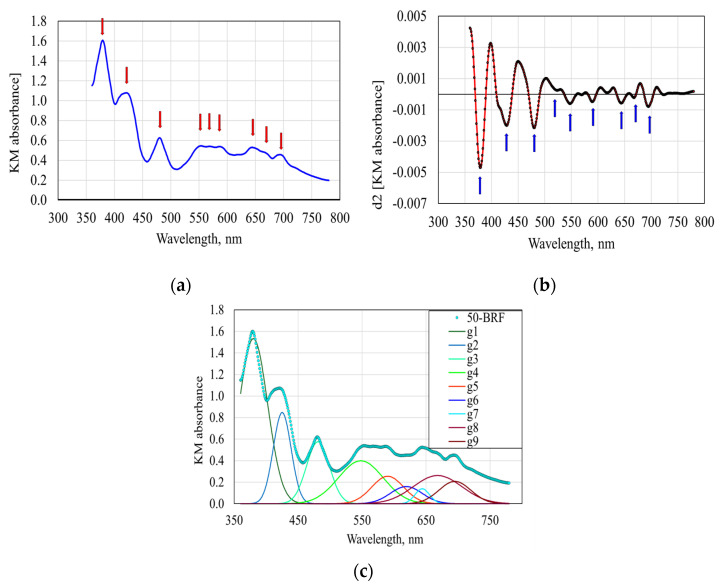
The deconvolution process of the KM spectra: (**a**) the variation in the KM absorbance with the wavelength; (**b**) the second derivative of the KM spectra; (**c**) the Gaussian distribution corresponding to the nine classes of optically active compounds in the 50-BRF sample.

The first step involved identifying the wavelengths assigned to the optically active compounds contained in the mixture. The background noise was eliminated, and the second-order derivative was found by applying Savitzky–Golay filtering (3rd-order polynomial, 25-point window). The centers of the individual distributions corresponded to the recorded minimum values. This analysis identified nine optically active components corresponding to wavelengths of 380, 429, 481, 548, 590, 619, 644, 668 and 695nm, respectively, with an accuracy of ±1 nm. Figure 4a presents the variation in the KM absorbance with the wavelength, while Figure 4b shows the second derivative. The maximum values of the peaks in Figure 4a correspond to the nine minimum values of the second derivative from Figure 4b.

In the second step, which represents the deconvolution process, for each wavelength determined above, a Gaussian distribution was considered, characterized by a specific amplification factor and a specific value of the standard deviation, which was initialized with value 1 in the iterative deconvolution process. For each wavelength in the range 360–780 nm, with a resolution of 1 nm, the absorbance was calculated as the sum of absorbances of the individual Gaussian components, and the sum of the squared error (SSE) was determined. By applying the Solver function in Microsoft Office Excel and using the solving method for nonlinear optimization Generalized Reduced Gradient, the optimal values of the multiplication factors and deviations of the considered distribution were calculated to ensure the minimum values of SSE. These values correspond to the distribution areas considered to be characteristic of the colored (optically active) components in the flour samples. In all cases, after obtaining the optimal deconvolution variant, the SSE values and the correlation coefficient between the base spectrum and the sum spectrum of the individual distributions were recorded.

Figure 4c shows the Gaussian distribution corresponding to the nine classes of optically active compounds in the 50-BRF sample. For all flour samples, the correlation coefficients between the base spectrum and the spectrum obtained as the sum of the individual distributions ranged from 0.9900 to 0.9942, while the sum of the squared error values ranged from 0.3264 to 1.4142, indicating a high accuracy of the results.

Table 4 shows the area values of the components identified after the deconvolution process. Their values increased with the BRF ratio increase in the flour mixtures. The lowest areas values were obtained for wavelengths of 619 nm and 644 nm, suggesting small amounts of corresponding colored compounds in WRF, 25-BRF and 75-BRF. The areas of nine individual distributions of optically active compound groups identified in WRF, BRF and their mixtures were proportional with their concentrations. Based on the results of the chemical analysis (Table 3a,b), in which four proanthocyanins and seven phenolic acids were identified, the correlation between their concentrations and the areas was established. For this purpose, multiple linear regression (SLR) was applied for a number of 9 × (4 + 7) = 99 models. The dependent variable was successively assimilated with the values of the concentrations of protoanthocyanins and phenolic acids in the analyzed flours, and the independent variable was successively assimilated with the corresponding values of the areas determined through spectral deconvolution analysis. In each case, the regression model was subjected to analysis using the ANOVA variant and the next quality parameters were calculated: the coefficient of determination (R^2^, square of the correlation coefficient between the observed dependent variable and the modeled variable), the value of the standard error of estimate (EEA), the mean percentage error (ER,%) and the statistical probability level. The predictive power of each model was evaluated using a cross-validation method, where the quality parameters were also determined. 

Several conclusions were drawn from the results:

(i) With the exception of area A# 6, assigned to the wavelength at 619 nm, the concentrations of four proanthocyanins correlate significantly (*p* < 0.05) with the areas resulting from the deconvolutions. The best correlation is registered in the case of area A# 9 (695 nm), for which the values of R^2^ were in the range of 0.9905 to 0.9977, the values of ER(%) were in the range of 3.0616–5.1745% and the predictive power of the model ranged from 5.37% to 8.31%.
(ii) The literature indicates the presence of anthocyanins around 627 nm [39], but the model did not assign a compound to the A#6, probably due to its low values of the areas associated with the Gaussian distributions (Table 4).
(iii) The concentration of protocatechuic acid significantly correlates (*p* < 0.00007) with the area A#2 assigned to the wavelength 429 nm, with values of 0.9970, 5.2081% and 5.5689% for the R^2^, ER (%) and predictive power, respectively.
(iv) The concentration of vanillic acid significantly correlates (*p* < 0.00004) with area A#9 (695 nm), with values of R^2^, ER (%) and predictive power of 0.9979, 2.7378% and 4.3299%.
(v) The syringic acid concentration is statistically correlated (*p* < 0.0219) with the area A#4 assigned to the wavelength at 548 nm, with an R^2^ of 0.8651, ER (%) of 14.6277% and predictive power of 24.7955%.
(vi) The concentrations of p-coumaric acid and ferulic acid are not significantly correlated to any of the nine areas resulting from the deconvolution process. The minimum values of the statistical probability level were 0.2138 and 0.3704, respectively. The positioning of the absorption maximum values for coumaric acid at 211, 225 and 310 nm and for ferulic acid at 275 and 326 nm [5] outside the analyzed spectral range explains the lack of correlations.
(vii) The concentrations of sinapic acid and ellagic acid both correlate significantly (*p* < 0.0116 and *p* < 0.0002) with area A#1 assigned to the wavelength of 380 nm with values of 0.9111 and 0.9950 for the R^2^, 3.8234% and 3.8730% for the ER (%) and a predictive power of 6.9287% and 7.5309%, respectively.

**Table 4:** The areas of optically active components in the rice flours and their wavelengths assignment after the deconvolution process.

Areas Associated with the Gaussian Distributions (g1–g9) in Figure 4c	Wavelengths Associated with the Gaussian Distributions, nm	Wavelength-Assigned Compounds from the Application of Multiple Linear Regression (SLR) to Deconvoluted Reflectance Spectra	Wavelengths of Maximum Absorption of Compounds Reported in the Literature	The Values of the Areas Associated with the Gaussian Distributions (g1–g9) in Figure 4c				
				WRF	25-BRF	50-BRF	75-BRF	BRF
A#1 associated with g1	380	Proanthocyanins	Peonidin 3-*O*-glucoside, 330 nm [5] Malvidin 3-*O*-glucoside, 348 nm [5]	38.45 ± 0.61 ^e^	59.45 ± 0.41 ^d^	84.44 ± 1.34 ^c^	105.29 ± 0.43 ^b^	128.12 ± 2.78 ^a^
		Sinapic acid	238 nm, 318 nm [5]					
		Ellagic acid	256 nm, 360–368 nm [5]					
A#2 associated with g2	429	Proanthocyanins	Malvidin 3-*O*-glucoside, 421 nm [5]	16.42 ± 0.27 ^e^	22.99 ± 0.27 ^d^	28.70 ± 0.92 ^c^	33.83 ± 0.17 ^b^	39.82 ± 1.48 ^a^
		Protocatechuic acid	297 nm, 258 nm [5]					
A#3 associated with g3	481	Proanthocyanins	Cyanidin-3-(6-O-coumaroyl) glucoside, 441 nm [5]	3.72 ± 0.13 ^e^	13.22 ± 0.16 ^d^	24.92 ± 0.72 ^c^	35.29 ± 0.28 ^b^	53.65 ± 1.29 ^a^
A#4 associated with g4	548	Proanthocyanins	Cyanidin-3-*O*-glucoside, 516 nm [5]	2.28 ± 0.10 ^e^	12.34 ± 0.08 ^d^	26.05 ± 0.77 ^c^	29.11 ± 0.09 ^b^	47.59 ± 1.28 ^a^
			Peonidin 3-*O*-glucoside, 517 nm [5]					
			Petunidin 3-*O*-glucoside, 526 nm [5]					
			Malvidin 3-*O*-glucoside, 528 nm [5]					
		Syringic acid	Co-pigments based on syringic acid, 550 nm [40]					
A#5 associated with g5	590	Proanthocyanins	Peonidin-3-(6-*O*-coumaroyl)glucoside, 522 nm Petunidin-3-(6-*O*-coumaroyl)glucoside, 534 nm Malvidin-3-(6-O-coumaroyl)glucoside, 534 nm Malvidin-3-(6-O-ca_eoyl)glucoside, 532 nm Malvidin-3-O-rutinoside, 526 nm [5]	0.47 ± 0.03 ^e^	6.71 ± 0.06 ^d^	10.64 ± 0.19 ^c^	19.10 ± 0.24 ^b^	26.54 ± 0.66 ^a^
A#6 associated with g6	619	-	Anthocyanins, 627 nm [39]	0	0	4.10 ± 0.22 ^b^	0	7.84 ± 0.31 ^a^
A#7 associated with g7	644	Proanthocyanins	Anthocyanins, 679 nm [41] Protein-tannin complexes, 679 nm [41] Chlorophyll *a* [42]	0.15 ± 0.01 ^e^	1.81 ± 0.03 ^d^	3.75 ± 0.10 ^b^	7.30 ± 0.06 ^b^	8.49 ± 0.19 ^a^
A#8 associated with g8	668	Proanthocyanins		0.63 ± 0.04 ^e^	12.95 ± 0.22 ^d^	14.48 ± 0.24 ^c^	24.08 ± 0.45 ^b^	36.25 ± 0.95 ^a^
A#9 associated with g9	695	Proanthocyanins	Methylated anthocyanins, 717 nm [43] Acylated anthocyanins, 717 nm [43] Protein-tannin complexes, 717 nm [43]	13.10 ± 0.70 ^e^	38.41 ± 0.41 ^d^	57.39 ± 2.50 ^c^	83.68 ± 1.36 ^b^	94.76 ± 3.84 ^a^
		Vanillic acid	Co-pigments based on vanillic acid, 540 nm [44]					
Sum of areas g1–g9				75.22	167.88	254.47	337.68	443.06

Results are presented as mean values ± standard deviations (n ≥ 3); different letters within the same row indicate significant differences (*p* < 0.05) between mean values (Tukey test). WRF—100% brown rice flour, 25-BRF—flour mixture with 25% BRF and 75% WRF, 50-BRF—flour mixture with 50% BRF and 50% WRF, 75-BRF—flour mixture with 75% BRF and 25% WRF, BRF—100% black rice flour.

As shown in Table 4, both proanthocyanins and phenolic acids associated with wavelengths in the range of 380–590 nm and 695 nm exhibited a strong bathochromic shift to higher wavelengths, compared to the values reported in the literature, which may be due to co-pigmentation with other classes of polyphenols [40]. In contrast, anthocyanins, protein–tannin complexes, methylated anthocyanins and acylated anthocyanins associated with wavelengths at 619, 644 and 668 nm exhibited a hypsochromic effect by shifting the wavelengths to lower values. The largest bathochromic shift was expressed by vanillic acid from the wavelength of 292 nm of the pure compound presented in the literature [5] to the wavelength of 695 nm. The work of Chen et al. [44] indicates an absorption wavelength around 510 nm for mulberry anthocyanin co-pigmented with vanillic acid in a ratio of 1:2.5 at 20 °C and pH = 3. By increasing the mulberry-vanillic acid ratio to 1:20, the wavelength shifted to around 540 nm, with the addition of co-pigments resulting in the bathochromic effect. In our work, the high concentration of vanillic acid (Table 3), expressed in large values for A#9 associated with the Gaussian distribution g9 (Table 4), could explain this large shift. The syringic acid also showed a large bathochromic shift, from the 276 nm absorption wavelength of the individual compound [5] to 548 nm (Table 4), which is explained by co-pigmentation with other polyphenols. The absorbance of around 550 nm for the bayberry anthocyanin–syringic acid co-pigment at a ratio of 1:30 was reported in the work of Zhu et al. [40].

The evolution of areas of peaks assigned to the nine colored groups in the rice flours (Table 4) confirms the results of the chemical analysis (Table 3). The lowest area value of 75.22 was obtained in the case of WRF, while the highest area value of 443.06 was obtained for WRF, corresponding to the lowest value of the total amount of phenolic acids and proanthocyanins (7.86 µg/g) in WRF and the highest value for the total amount of phenolic acids and proanthocyanins (963.94 µg/g) in BRF. There are no studies available in the literature dealing with the identification of optically active compound groups in rice flour, based on deconvolution of the reflectance spectra.

## 4. Conclusions

This study provides the characterization of the composition, starch and protein structure of brown rice flour, black rice flour and their mixtures in proportions of 25%, 50% and 75%. The proximate composition results indicated that black rice flour is a higher source of nutrients than brown rice flour, in terms of proteins, total minerals, lipids, crude fiber, polyphenols, flavonoids and proanthocyanins. All four proanthocyanins and five of seven phenolic acids were significantly correlated with one of the nine optically active compound groups differentiated from the processed reflectance spectra. This proved that the reflectance spectra can be used for the semi-quantitative analysis of polyphenols by revealing the optically active compound groups in the sample and to evidence the co-pigmentation that occurred between anthocyanins themselves or with other molecules. 

When recommending a specific type of flour or flour mixture for product development, several aspects should be taken into account. From a nutritional perspective, considering the proximate analysis, a greater variety and concentrations of polyphenols and a lower glycemic index, black rice flour is strongly recommended when developing gluten-free-based products. From a technological perspective, given the more ordered starch structure in black rice flour, higher cooking temperatures can be expected and/or longer exposure times, and products have higher hardness and lower adhesiveness. The bioactive compounds in black rice-based products may be affected to a larger extent than in brown rice-based products due to the higher baking regime. Regardless of nutritional and technological considerations, the use of black rice flour in the production of functional foods may be limited by consumer acceptance. Therefore, the exclusive use of black rice flour or a mixture of brown rice flour and black rice flour should be investigated individually, for each food type. Further research is being conducted to investigate the properties of bread made from brown rice flour, black rice flour and their mixtures and their acceptance by consumers.

## Figures and Tables

**Table 1:** Characteristics of brown rice flour, black rice flour and their mixtures.

Parameter		Black Rice Flour Substitution, %				
		0 (Brown Rice Flour) WRF	25 25-BRF	50 50-BRF	75 75-BRF	100 (Black Rice Flour) BRF
Moisture, g/100 g		12.79 ± 0.32 ^a^	12.58 ± 0.41 ^a^	12.42 ± 0.11 ^a^	12.28 ± 0.20 ^a^	12.14 ± 0.29 ^a^
Protein, g/100 g		8.73 ± 0.18 ^d^	9.32 ± 0.11 ^c^	9.78 ± 0.12 ^c^	10.33 ± 0.15 ^b^	10.98 ± 0.28 ^a^
Total lipids, g/100 g		2.11 ± 0.02 ^e^	2.49 ± 0.02 ^d^	3.11 ± 0.03 ^c^	3.50 ± 0.04 ^b^	4.01 ± 0.11 ^a^
Ash, g/100 g		0.72 ± 0.01 ^e^	0.83 ± 0.01 ^d^	1.01 ± 0.01 ^c^	1.18 ± 0.01 ^b^	1.36 ± 0.02 ^a^
Crude fiber, g/100 g		2.54 ± 0.08 ^e^	3.11 ± 0.06 ^d^	3.82 ± 0.09 ^c^	4.39 ± 0.14 ^b^	5.11 ± 0.18 ^a^
Total starch, g/100 g		71.08 ± 3.38 ^a^	70.03 ± 2.39 ^a^	68.98 ± 2.84 ^a^	67.93 ± 2.08 ^a^	66.87 ± 2.25 ^a^
Carbohydrates, g/100 g		85.90 ± 4.3^a^	84.25 ± 2.90 ^a^	82.28 ± 3.92 ^a^	80.60 ± 3.29 ^a^	78.54 ± 1.78^a^
Energy, Kcal/100 g		402.59 ± 16.71 ^a^	402.91 ± 9.55 ^a^	403.87 ± 5.78 ^a^	404.00 ± 8.56 ^a^	404.39 ± 12.58 ^a^
Amylose content, %		19.16 ± 0.70 ^c^	20.11 ± 0.45 ^bc^	20.92 ± 0.24 ^ab^	21.46 ± 0.20 ^ab^	22.30 ± 0.99 ^a^
Polyphenols, mg GAE/100 g	Free polyphenols	93.13 ± 3.41 ^e^	105.34 ± 4.43 ^d^	117. 23 ± 2.78 ^c^	131.13 ± 1.60 ^b^	142.42 ± 3.46 ^a^
	Bound polyphenols	54.63 ± 2.47 ^e^	93.68 ± 3.89 ^d^	132.80 ± 2.88 ^c^	169.65 ± 5.19 ^b^	209.79 ± 7.34 ^a^
	Total polyphenols	147.76 ± 4.09 ^e^	199.02 ± 8.14 ^d^	250.03 ± 8.33 ^c^	300.78 ± 9.35 ^b^	352.21 ± 9.72 ^a^
Total flavonoids, mg_QE_/g		0.41 ± 0.01 ^e^	0.90 ± 0.02 ^d^	1.46 ± 0.02 ^c^	2.33 ± 0.06 ^b^	3.47 ± 0.09 ^a^
Total proanthocyanins, mg_C_/g		0.01 ± 0.00 ^e^	0.38 ± 0.00 ^d^	0.74 ± 0.00 ^c^	1.16 ± 0.01 ^b^	1.49 ± 0.04 ^a^
CIEL*a*b* coordinates	L*	89.31 ± 0.27 ^a^	76.15 ± 0.09 ^b^	69.60 ± 0.33 ^c^	66.02 ±0.05 ^d^	60.72 ± 0.34 ^e^
	a*	−7.77 ± 0.06 ^e^	−6.31 ± 0.03 ^d^	−5.23 ± 0.02 ^c^	−4.80 ± 0.02 ^b^	−3.96 ±0.03 ^a^
	b*	15.05 ± 0.17 ^a^	6.74 ± 0.03 ^b^	4.77 ± 0.05 ^c^	3.78 ± 0.3 ^d^	2.72 ± 0.05 ^e^
RGB coordinates	R	11.19 ± 0.11 ^a^	7.09 ± 0.019 ^b^	5.67 ±0.07 ^c^	4.96 ± 0.01 ^d^	4.07 ±0.06 ^e^
	G	10.93 ± 0.07 ^a^	8.35 ±0.027 ^b^	6.91 ± 0.08 ^c^	6.19 ± 0.01 ^d^	5.16 ±0.064 ^e^
	B	13.72 ± 0.10 ^a^	9.27 ±0.03 ^b^	7.43 ±0.09 ^c^	6.54 ±0.01 ^d^	5.35 ± 0.07 ^e^
Images of flour samples		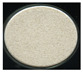	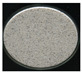	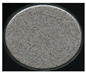	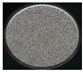	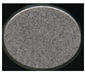

Results are presented as mean values ± standard deviations (n ≥ 3); different letters within the same row indicate significant differences (*p* < 0.05) between mean values (Tukey test). WRF–100% brown rice flour, 25-BRF–flour mixture with 25% BRF and 75% WRF, 50-BRF–flour mixture with 50% BRF and 50% WRF, 75-BRF–flour mixture with 75% BRF and 25% WRF, BRF–100% black rice flour.

**Table 2:** Features of the protein secondary structure and starch bands in the brown rice flour (WRF), black rice flour (BRF) and flour mixtures resulting from FTIR analysis.

Flour Type	Protein Secondary Structure Analysis (%)				Starch Bands	
	α-Helix	β-Sheet	β-Turns	Random Coil	R_1047/1022_	R_995/1022_
WRF	14.22 ± 0.72 ^e^	30.52 ± 1.26 ^a^	18.80 ± 0.41 ^a^	36.46 ± 0.89 ^a^	1.12 ± 0.03 ^d^	1.32 ± 0.05 ^c^
25-BRF	15.72 ± 0.65 ^d^	31.01 ± 1.45 ^a^	16.66 ± 0.41 ^b^	36.61 ± 1.15 ^a^	1.17 ± 0.06 ^cd^	1.38 ± 0.06 ^bc^
50-BRF	17.38 ± 0.37 ^c^	31.44 ± 0.98 ^a^	14.42 ± 0.28 ^c^	36.76 ± 1.62 ^a^	1.22 ± 0.03 ^bc^	1.45 ± 0.04 ^ab^
75-BRF	18.92 ± 0.57 ^b^	31.89 ± 1.30 ^a^	12.30 ± 0.37 ^d^	36.89 ± 1.80 ^a^	1.27 ± 0.03 ^ab^	1.51 ± 0.05 ^a^
BRF	20.47 ± 0.23 ^a^	32.40 ± 0.93 ^a^	10.12 ± 0.27 ^e^	37.01 ± 1.88 ^a^	1.32 ± 0.02 ^a^	1.57 ± 0.03 ^a^

Results are presented as mean values ± standard deviations (n ≥ 3); different letters within the same column indicate significant differences (*p* < 0.05) between mean values (Tukey test); WRF–100% brown rice flour, 25-BRF–flour mixture with 25% BRF and 75% WRF, 50-BRF–flour mixture with 50% BRF and 50% WRF, 75-BRF–flour mixture with 75% BRF and 25% WRF, BRF–100% black rice flour.

**Table 3:** (**a**) Proanthocyanins identified in the brown rice flour (WRF), black rice flour (BRF) and their mixtures. (**b**) Phenolic acids identified in the brown rice flour (WRF), black rice flour (BRF) and their mixtures.

(**a**)								
**Sample**	**Proanthocyanins, µg/g**							
	**Cyanidin-3-*O*-glucoside**		**Peonidin-3-*O*-glucoside**	**Malvidin 3-*O*-glucoside**		**Petunidin Chloride**		**Total Amount of Proanthocyanins**
WRF	Nd		nd	nd		nd		-
25-BRF	130.23 ± 5.25 ^d^		68.07 ± 7.12 ^d^	1.60 ± 0.18 ^d^		0.35 ± 0.03 ^d^		200.25
50-BRF	265.47 ± 7.81 ^c^		14.34 ± 2.37 ^c^	2.82 ± 0.08 ^c^		0.59 ± 0.02 ^c^		283.22
75-BRF	441.19 ± 1.56 ^b^		203.74 ± 5.66 ^b^	4.62 ± 0.38 ^b^		0.88 ± 0.01 ^b^		650.43
BRF	481.68 ± 7.63 ^a^		226.33 ± 0.06 ^a^	5.60 ± 0.04 ^a^		1.07 ± 0.05 ^a^		714.68
(**b**)								
**Sample**	**Phenolic Acids, µg/g**							
	**Protocatechuic Acid**	**Vanillic Acid**	**Syringic Acid**	**p-Coumaric Acid**	**Ferulic** **Acid**	**Sinapic Acid**	**Ellagic Acid**	**Total Amount of Phenolic Acids**
WRF	nd	0.27 ± 0.00 ^e^	1.13 ± 1.13 ^c^	1.03 ± 0.01 ^ab^	4.84 ± 0.03 ^a^	0.34 ± 0.01 ^c^	0.25 ± 0.03 ^e^	7.86
25-BRF	54.92 ± 4.40 ^d^	7.36 ± 0.34 ^d^	3.48 ± 0.00 ^b^	1.02 ± 0.01 ^b^	3.13 ± 0.00 ^c^	0.32 ± 0.01 ^c^	1.75 ± 0.04 ^d^	71.98
50-BRF	105.54 ± 3.11 ^c^	14.43 ± 0.34 ^c^	4.81 ± 0.28 ^ab^	1.09 ± 0.02 ^ab^	3.29 ± 0.05 ^c^	0.42 ± 0.01 ^b^	3.69 ± 0.08 ^c^	133.27
75-BRF	162.18 ± 4.62 ^b^	22.22 ± 0.28 ^b^	3.84 ± 0.07 ^b^	1.13 ± 0.03 ^a^	3.60 ± 0.02 ^b^	0.46 ± 0.04 ^ab^	5.06 ± 0.09 ^b^	198.49
BRF	205.80 ± 1.71 ^a^	25.07 ± 1.06 ^a^	6.03 ± 0.14 ^a^	0.89 ± 0.03 ^c^	3.59 ± 0.04 ^b^	0.51 ± 0.05 ^a^	7.37 ± 0.19 ^a^	249.26

Results are presented as mean values ± standard deviations (n ≥ 3); different letters within the same column indicate significant differences (*p* < 0.05) between mean values (Tukey test). nd—not detected; WRF–100% brown rice flour, 25-BRF–flour mixture with 25% BRF and 75% WRF, 50-BRF–flour mixture with 50% BRF and 50% WRF, 75-BRF–flour mixture with 75% BRF and 25% WRF, BRF–100% black rice flour.

## Data Availability

The original contributions presented in the study are included in the article/Supplementary Material, further inquiries can be directed to the corresponding author.

## Supplementary Materials

The following supporting information can be downloaded at: https://www.mdpi.com/article/10.3390/foods13111592/s1 [19,45,46,47,48,49,50,51], Figure S1: The experimental results of reference starch digestion (a), reference glucose release (b), starch digestion from rice flour (c), and glucose release from rice flours bread (d). Figure S2: The fit of the first-order kinetic model to the experimental results of glucose (a) and starch (b) digestion from rice flours. Table S1: Selected FTIR frequencies and their peak assignment for the spectra. Table S2: The absorbance ratios for lipids, proteins and polysaccharides extracted from FTIR spectra of brown rice flour and black rice flour. Table S3: The parameters of first-order kinetic model applied to describe the in vitro starch digestion, the digested starch fractions and estimated glycemic index of rice flours and flour.
