# Supplementary material for: Characterization of Polyphenol Composition and Starch and Protein Structure in Brown Rice Flour, Black Rice Flour and Their Mixtures
Source: Foods. 2024 May 21;13(11):1592. doi: 10.3390/foods13111592 (PMC11172181; doi:10.3390/foods13111592)
Supplement: Supplementary file 1 [file foods-13-01592-s001.zip › foods-3007348-supplementary.pdf]

Supplementary Material

Characterization of Polyphenol Composition and Starch and Protein Structure in Brown Rice Flour, Black Rice Flour and Their Mixtures

Alexandra Uivarasan [1], Jasmina Lukinac [2], Marko Jukic [2], Gordana Šelo [2], Anca Peter [1], Camelia Nicula [1], Anca Mihaly Cozmuta [1] and Leonard Mihaly Cozmuta [1,*]

[1] Department of Chemistry-Biology, Technical University of Cluj Napoca, 430122 Baia Mare, Romania; uivarasan.al.alexand@student.utcluj.ro (A.U.); anca.peter@cb.utcluj.ro (A.P.); camelia.nicula@cb.utcluj.ro (C.N.); mihaela.mihaly@cb.utcluj.ro (A.M.C.)

[2] Faculty of Food Technology, Josip Juraj Strossmayer University of Osijek, 31000 Osijek, Croatia; jlukinac@ptfos.hr (J.L.); marko.jukic@ptfos.hr (M.J.); gordana.selo@ptfos.hr (G.Š.)

* Correspondence: mihalyleonard@yahoo.com; Tel.: +40-741-949-669

2.SM. Materials and methods

2.1.SM. Dry matter content

Dry matter content was measured gravimetrically by drying the homogenized samples at 105°C to constant weight (Binder GmbH, Germany).

2.2.SM. Hydration property of rice flours

The water retention capacity of the studied flours was measured according to AACC Method 56-11.02 (2000). Accurately weighed 5 g of flour sample was hydrated in a 50 mL centrifuge tube with 25.0 mL of water for 20 min until no lumps were observed. The suspension was centrifuged at 1000 × g for 15 min. After discarding the supernatant and emptying the tube for 10 min, the weight of the swollen sample was measured. The sample water retention capacity (WRC) was calculated based on the initial weight of the sample and expressed in g/g.

2.3.SM. *In vitro* glycemic index analysis

In vitro digestions of rice flours were performed in triplicate considering the sum of oral-gastric-intestinal stages, according to the INFOGEST protocol (Brodkorb et al., 2019).

The method proposed by Yusufoğlu et al. (2021) adapted to our work was used to calculate the glycemic index of samples. Intestinal digestate samples were collected every 5 min for the first 30 min and every 10 min in the range of 30 to 180 min. They were placed in an ice bath to inactivate amylase and centrifuged at 6,000 × *g* for 30 min to separate the supernatant. The digestion was initiated in individual tubes for each sampling time, to ensure higher reproducibility of the results. To measure the amount of glucose, a volume of 10 μL of supernatant was mixed with 1 mL of glucose oxidase/peroxidase/4-aminoantipirin/phenol solution, and the decomposition of glucose to the chinonimine red compound was measured. After 10 min of incubation at 37°C, the absorbance of the solution was determined against the blank value by spectrophotometry at 500 nm (BTS-350 analyzer, Biosystems, Spain). Blank digestion was also conducted following the same protocol. The glucose concentration (mg/g sample) was calculated at each sampling time and the result was quantified as the difference between the amount of glucose in the sample and the blank digestion. Glucose concentrations were plotted against sampling times between 0 and 180 min (hydrolysis curve) and the area under the hydrolysis curve ($AUHC_S$) was calculated via the Excel Program.

In vitro digestion was also performed using glucose as a reference (formulation is provided in Supplementary Material). After determining the hydrolysis curve and the area under the hydrolysis curve ($AUHD_R$), the Hidrolisys index (HI) of glucose in samples was calculated according to the Eq. 4 (Klunklin et Savage, 2018):

$$HI = AUHC_R \cdot 100/AUHC_S, \qquad \text{(Eq. 3.SM)}$$

where: $AUHC_S$ and $AUHC_R$ represent the area under the hydrolysis curve for samples and reference, respectively.

The estimated Glycemic index (eGI) of the samples was calculated using Eq. 5 (Goni et al., 1997):

$$eGI = 39.71 + 0.549 \cdot HI \qquad \text{(Eq. 4.SM)}$$

2.4.SM. Starch digestibility

Based on the profiles of glucose release during digestion, starch digestibility was assessed. Depending on the rate of hydrolysis to glucose under the action of α-amylase and absorption in the gastrointestinal tract, starch is classified in three fractions: (i) Rapidly digestible starch (RDS) hydrolyzes within the first 20 min, which leads to a rapid release of glucose from the small intestine into the blood stream; (ii) Slow digestible starch (SDS) hydrolyzes between 20 and 120 min, resulting in prolonged glucose release into the blood stream; and (iii) Resistant starch (RS), which resists digestion and absorption in the small intestine and is available for fermentation through glycolysis by microorganisms in the colon to short chain fatty acids. The fractions of RDS(%) and SDS(%) in the samples were calculated according to the Eqs. 5 and 6 (Xiang et al., 2023):

$$RDS\ (\%) = (G_{20} - G_{0}) \cdot 0.9 \times 100/TS \qquad \text{(Eq. 5.SM)}$$

$$SDS\ (\%) = (G_{120} - G_{20}) \cdot 0.9 \times 100/TS \qquad \text{(Eq. 6.SM)}$$

where: G_0 is the free glucose content of the sample, mg/g sample; G_{20} is the glucose released within 20 min, mg/g sample; G_{120} is the glucose released within 120 min, mg/g sample; TS is the total starch content of the sample, mg/g sample; 0.9 is the conversion factor of glucose into starch.

The digestion graph was plotted as the ratio of total digested starch (%) to digestion time (min). Experimental results were used to fit the kinetic models of starch digestion, considering the first-order kinetic model for starch hydrolysis (Tang et al., 2021):

$$C = C_{\infty} (1 - e^{-kt}) \qquad \text{(Eq. 7.SM)}$$

where C is the ratio of starch hydrolyzed at time t, %; C_{∞} is the estimated ratio of starch hydrolyzed in the end of reaction (%); k is the kinetic constant, min^{-1} and t is the sampling time, min.

3.SM. Results and discussions

3.1.SM. Water retention capacity

Significant differences ($p < 0.05$) between hydration capacity between WRF and BRF can be observed in Table 1.SM. The water retention capacity of BRF is 1.14-times higher than that of WRF. The water binding capacity of flour mixtures increased proportionally with the ratio of black rice

flour. With an amylose content of more than 20%, the BRF is classified as a medium-amylose content, while the WRF with 19.16% is classified as a low-amylose content. The amylose concentration is 16.39% higher in BRF than in WRF. The result is far higher than the value of 6.47% reported by Farooq et al (2021), whereby parameters such as variety, growth or processing conditions could be considered responsible. In our study, the Pearson coefficient of r = 0.97 indicates a strong positive correlation between WRC and amylose content in flours and flour mixtures, which is stronger than the value r = 0.72 reported by Gui et al. (2021).

Table 1.SM. Water retention capacity for brown rice flour, black rice flour and their mixtures

Water retention capacity, g/g	2.11 ± 0.03^{c}		2.19 ± 0.04^{bc}	2.22 ± 0.04^{abc}	2.35 ± 0.10^{ab}	2.40 ± 0.12^{a}

3.2.SM. *In vitro* starch digestion and estimated glycemic index

Figures 1.a.SM to 1.d.SM show the experimental results of starch digestion and released glucose in the rice flours, as well as reference glucose and starch. Overall, digestion in the first 20 minutes, which was assumed to be from the RDS fraction, occurred at the highest level for the reference sample and at lower levels for rice flours. After 20 minutes, digestion gradually slowed until it reached a plateau. For the rice flours and their mixtures, the amounts of digested starch and released glucose decreased with the degree of substitution of WRF by BRF, the higher the ratio of BRF the lower the amounts of starch digested, and glucose released.

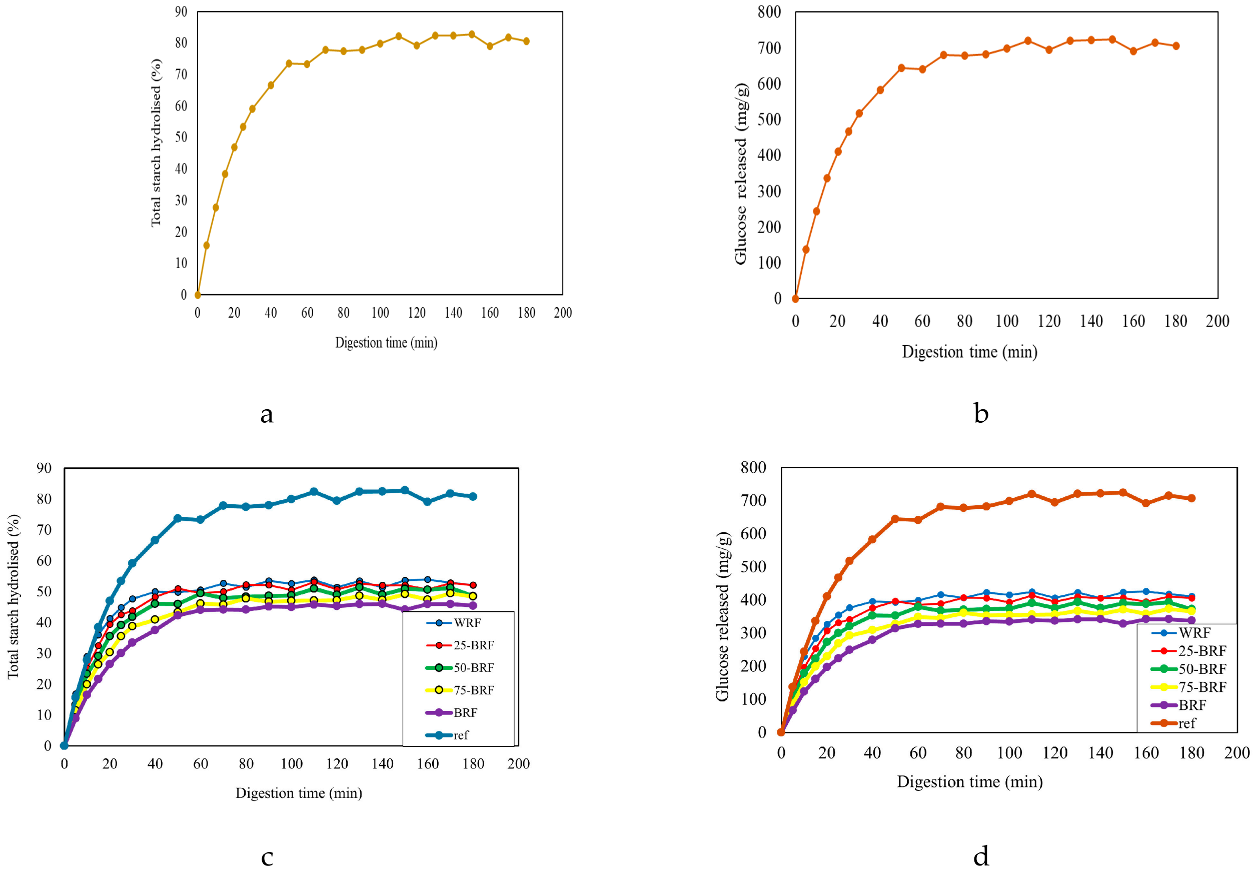

Figure 1.SM. The experimental results of reference starch digestion (a), reference glucose release (b), starch digestion from rice flour (c), and glucose release from rice flours bread (d).

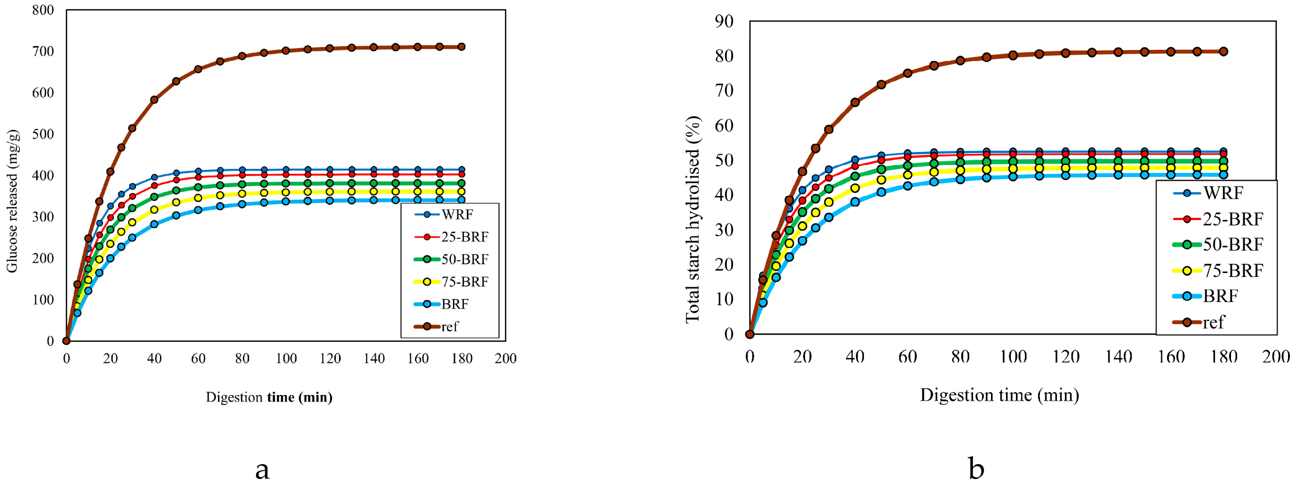

Figure 2.SM. The fit of the first-order kinetic model to the experimental results of glucose (a) and starch (b) digestion from rice flours

Fitting the first-order kinetic model to the experimental digestion of starch and glucose from rice flours with an accuracy of $R^2 > 0.92$, resulted in Figures 2.a.SM and 2.b.SM, and the parameters extracted from the kinetic model are shown in Table 4. The highest values for the maximum digestion extent $C(\infty)$ were observed for brown rice flour and decreased with the addition of black rice flour. Farooq et al. (2021) reported values of 47.9% and 52.7% for $C(\infty)$ in brown rice and black rice, and values of 0.036 min^{-1} and 0.040 min^{-1} for associated kinetic constants. The digestion method and mathematical model used may explain the differences.

The addition of black rice flour in the flour mixtures resulted in significantly lower ($p < 0.05$) RSD values and higher SDS values than in the brown rice flour. For example, adding 75% black rice flour reduces the RDS content in the flour mixture by 24.81% while increasing the SDS content by 33.47%. The results showed a reduction in starch digestibility and released glucose with the addition of black rice flour, which is consistent with the conclusions of the FTIR analysis. As shown in Table 2.SM, the estimated glycemic index (eGI) significantly decreased ($p<0.05$) with increasing the proportion of the BRF substituted in the flour mixtures. Digestion of BRF resulted in an eGI of 70.80 compared to 77.52 in the case of WRF. The reduction in starch digestion by the addition of black rice flours may be related to the higher amount of amylose, lipids, and proteins in BRF compared to WRF. The presence of amylose-amylose, amylose-lipids, and amylose-proteins complexes (Xiang et al., 2023) strengthens the starch structure. By creating a complex effect on the spatial arrangement, the diffusion of α-amylase and water to the starch granules is restricted, resulting in a reduction in starch sensitivity during digestion and subsequently an increase in the SDS ratio. Furthermore, a more disordered starch structure in WRF compared to BRF, expressed in lower values of $R_{1047/1022}$ and $R_{995/1022}$ indices (Table 3) suggests a higher sensitivity to starch digestion in WRF. The higher crude fiber content in BRF also promotes the slowing down of starch digestion. Strong negative correlations between amylose and eGI ($r = -0.98$), $R_{1047/1022}$ and eGI ($r = -0.98$), $R_{995/1022}$ and eGI ($r = -0.98$), associated with strong positive correlations between amylose and SDS ($r = 0.98$), $R_{1047/1022}$ and SDS ($r = 0.98$) and $R_{995/1022}$ and SDS ($r = 0.98$) support our results.

Table 2.SM. The parameters of first-order kinetic model applied to describe the *in vitro* starch digestion, the digested starch fractions and estimated glycemic index of rice flours and flour mixtures

Sample code	C(∞), %	k, min^{-1}	HI	eGI	RDS(20 min), %	SDS(20-180 min), %	RS(>180 min), %
Flours and flour mixtures							
WRF	52.51 ± 0.82^{a}	0.08 ± 0.00^{a}	68.88 ± 2.83^{a}	77.52 ± 0.89^{a}	41.36 ± 0.97^{a}	11.15 ± 0.14^{e}	47.49 ± 1.43^{c}
25-BRF	51.77 ± 1.10^{a}	0.07 ±0.00^{b}	67.15 ± 1.78^{ab}	76.58 ± 0.93^{ab}	38.39 ± 1.33^{b}	13.38 ± 0.29^{d}	48.23 ± 1.50^{c}
50-BRF	49.75 ± 1.00^{ab}	0.06 ± 0.00^{c}	63.92 ± 0.79^{bc}	74.80 ± 0.76^{bc}	35.12 ± 1.10^{c}	14.63 ± 0.44^{c}	50.25 ± 0.99^{bc}
75-BRF	47.86 ± 1.22^{bc}	0.05 ± 0.00^{d}	60.47 ± 1.22^{cd}	72.91 ± 1.22^{cd}	31.10 ± 1.25^{d}	16.76 ± 0.46^{b}	52.14 ± 1.44^{ab}
BRF	45.84 ± 1.42^{c}	0.04 ± 0.00^{e}	56.63 ± 1.77^{d}	70.80 ± 0.79^{d}	26.85 ± 0.64^{e}	18.99 ± 0.38^{a}	54.16 ± 1.39^{a}
R^2	0.9784	0.9947	0.9836	0.9836	0.9929	0.9923	0.9783

Results are presented as mean values ± standard deviations ($n \geq 3$);

Different letters within the same column indicate significant differences ($p < 0.05$) between mean values (Tukey test);

WRF – 100% brown rice flour,25-BRF – flour mixture with 25% BRF and 75% WRF, 50-BRF – flour mixture with 50% BRF and 50% WRF, 75-BRF – flour mixture with 75% BRF and 25% WRF, BRF – 100% brown black rice flour;

C(∞) – the maximum hydrolysis extents, %; k - the kinetic constant, min^{-1}; HI – hydrolysis index, adimensional; GI – estimated glycemix index, adimensional; RSD(20 min) – the ratio of rapidly digestible starch, %; SDS (20-180 min) – the ratio of slowly digestible starch, %; RS(>180 min) – the ratio of resistant starch, %.

3.3. FTIR analysis

Table 3.SM: Selected FTIR frequencies and their peak assignment for the spectra

**Band (cm^{-1})**	**Assignment**	**Reference**
	**OH region**	
3500-3000	O-H stretching vibration	Zhang et al. (2023)
	**Proteins region**	
1700-1600<br>(1652)	Amide I absorption<br>(C=O stretching)	Ying et al. (2017)
1632-1629	Amide I absorption<br>(N-H bending vibration)	
1575-1480<br>(1541)	Amide II absorption<br>(N-H bending vibration coupled to C-N stretching)	
1457-1449<br>(1455)	asymmetric CH_3 bending modes of the<br>methyl groups of proteins	

1390-1340	symmetric CH_3 bending modes of the methyl groups of proteins	
**Polysaccharides region**		
1370-1362 (1370)	CH_2 bending mode (xyloglican, cellulose)	Ying et al. (2017)
1335-1320 (1334)	CH deformation from ring vibration (polysaccharides, pectin, cellulose)	
1236	C-O stretching (pectin)	
1200-700<br>1200-880	starch region<br>carbohydrate region	
1160-1146 (1149)	O-C-O symmetric stretching (glycosidic link of cellulose, pectin)	
1060-960	C-O , C-C bending vibrations (specific band of starch)	Xiang et al. (2023)
1076-1075	C-O stretching and C-C stretching (xyloglucan)	Ying et al. (2017)
1019-1000 (1014)	C-O stretching and C-C stretching (pectin, cellulose)	
930-927	C-O stretching vibration (carboxylate groups)	Copikova et al. (2001)
860-858	C-O stretching vibration (pectine)	
**Lipids region**		

2924-2921 (2922)	methylene (CH_2) vibration from lipids asymmetric C-H stretching	Xiang et al. (2023)
2854-2850 (2851)	methylene (CH_2) vibration from lipids symmetric C-H stretching	
1745-1740 (1744)	C=O stretching of triglycerides C=O stretching of alkyl ester	Ying et al. (2017)
2924-2921 (2922)	methylene (CH_2) vibration from lipids asymmetric C-H stretching	Xiang et al. (2023)

Table 4.SM. The absorbance ratios for lipids, proteins and polysaccharides extracted from FTIR spectra of Brown rice flour and black rice flour

Wave number, cm^{-1}	Peak assignment	Absorbance in black rice flour	Absorbance in brown rice flour	Ratios of absorbances	Mean of individual ratios
2922	Lipids Ying et al. (2017)	0.5294	0.3432	1.5425	1.63 ± 0.13
2851		0.3536	0.2254	1.5688	
1744		0.302	0.169	1.7870	
1652	Proteins Ying et al. (2017)	0.2813	0.219	1.2845	
1455		0.2085	0.1778	1.1727	1.16 ± 0.05
1236		0.1656	0.1374	1.2052	

1149	Polysaccharides Ying et al. (2017)	0.4365	0.3973	1.0987	
1075		0.6001	0.5699	1.0530	1.05 ± 0.03
930		0.2557	0.2368	1.0798	
860		0.0962	0.094	1.0234	

References

AACC Method 56-11.02 (AACC, 2000) – Solvent retention capacity

AACC Method 76–13 - Assay for analysis of total starch in cereal products.

Annex XIV of EU Regulation No 1169/2011 of the European Parliament and of the Council, of 25 October 2011.

AOAC 945.18-B (1995). Kjeldahl's method for protein determination in cereals and feed. Determination of total fat in flour, bread, bakery product and pasta with preliminary acid hydrolysis. Determination of Ash in Animal Feed. UDK 127 Operating manual.

AOAC 920.39.C (1995). The Soxhlet method for Cereal Fat.

AOAC 936.07 (2013). Ash in flour.

Brodkorb A., Balance S., Bohn T., Bourlieu-Lacanal C., Boutrou R., Carrière F., Clemente A., Corredig M., Dupont D., Dufour C., Edwards C., Golding M., Karakaya S., Kirkhus B., Le Feunteun S., Lesmes U., Macierzanka A., Mackie A.R., Martins C., Marze S., McClements D.J., Ménard O., Minekus M., Portmann R., Santos C.N., Souchon I., Singh R.P., Vegarud G.E., Wickham M.S.J, Weitschies W., Recio I.(2019). INFOGEST static in vitro simulation of gastrointestinal food digestion. NATURE PROTOCOLS. https://doi.org/10.1038/s41596-018-0119-1.

Copikova J., Synytsya A., Cerna M., Kaasova J., Novotna M. (2001). Application of FT-IR spectroscopy in detection of food hydrocolloids in confectionery jellies and food supplements. Czech J. Food Sci, 19, 51 – 56.

Zhang G., Xuan Y., Lyu F, Ding Y (2023). Microstructural, physicochemical properties and starch digestibility of brown rice flour treated with extrusion and heat moisture. International Journal of Biological Macromolecules, 242, 124594.

Xiang G., Li J., Lin Q, Zhang Y., Ding Y., Guo X., Pan Q., Liu q., Fu X., Yang Y., Han W., Fang Y. (2023). The effect of heat-moisture treatment changed the binding of starch, protein and lipid in rice flour to affect its hierarchical structure and physicochemical properties. Food Chemistry: X, 19, 100785.

Ying D.Y., Hlaing M.M., Lerissona J., Pitts K., Cheng L., Sanguansri L., Augustin M.A. (2017). Physical properties and FTIR analysis of rice-oat flour and maize-oat flour based extruded food products containing olive pomace. Food Research International, 100, 665–673.
